# Predicting patients with dementia most at risk of needing psychiatric inpatient or enhanced community care using routinely collected clinical data: a retrospective multi-site cohort study

**DOI:** 10.1192/bjp.2024.14

**Published:** 2024-06

**Authors:** Sabina R. London, Shanquan Chen, Emad Sidhom, Jonathan R. Lewis, Emma Wolverson, Rudolf N. Cardinal, David Roalf, Christoph Mueller, Benjamin R Underwood

**Affiliations:** i University of Cambridge, Department of Psychiatry, Herchel Smith Building, Forvie Site, Robinson Way, Cambridge CB2 0SZ; ii Department of Psychiatry, Perelman School of Medicine, University of Pennsylvania, Philadelphia, PA, 19104; iii University of Cambridge, Department of Clinical Neurosciences, Level 3, A Block, Box 165, Cambridge Biomedical Campus, CAMBRIDGE, CB2 0QQ; iv Cambridgeshire and Peterborough NHS Foundation Trust, Windsor Unit, Fulbourn Hospital, Cambridge CB21 5EF, United Kingdom; v Faculty of Health Sciences, University of Hull, Hull, UK; vi Department of Psychological Medicine, Institute of Psychiatry, Psychology & Neuroscience, King’s College London, London, UK; vii South London and Maudsley NHS Foundation Trust, London, UK; viii International Centre for Evidence in Disability, London School of Hygiene & Tropical Medicine, London, United Kingdom, WC1E 7HT

## Abstract

**Background:**

Dementia is a common and progressive condition whose prevalence is growing worldwide. It is challenging for healthcare systems to provide continuity in clinical services for all patients from diagnosis to death.

**Aims:**

To test whether patients who are most likely to need enhanced support later in the disease course can be identified at the point of diagnosis, thus allowing the targeted intervention.

**Method:**

We used clinical information collected routinely in de-identified electronic patient records from two United Kingdom NHS Trusts to identify at diagnosis which patients were at increased risk of needing enhanced care (psychiatric inpatient or intensive (crisis) community care).

**Results:**

We examined the records of a total of 25,326 patients with dementia. A minority (16% in Cambridgeshire and 2.4% in London) needed enhanced care. Patients who needed enhanced care differed from those who did not in age, cognitive test scores, and Health of the Nation Outcome Scale scores. Logistic regression discriminated risk with an area under the receiver operating characteristic curve (AUROC) of up to 0.78 after 1 year and 0.74 after 4 years. We were able to confirm the validity of the approach in two Trusts which differed widely in the populations they serve.

**Conclusions:**

It is possible to identify, at the time of diagnosis of dementia, patients most likely to need enhanced care later in the disease course. This permits the development of targeted clinical interventions for this high-risk group.

## Background

The syndrome of dementia involves a progressive loss of cognitive ability and gradually increasing impairment of function which ultimately leads to death. Dementia is already a significant problem with an estimated 55 million sufferers worldwide, and a growing problem with a predicted 139 million people living with dementia by 2050 (1). An estimated 60% of current cases are in low- and middle-income countries, but even in high-income countries the number of patients and the level of their disability means that healthcare systems struggle to meet demand. For example, in the United Kingdom specialist dementia services often struggle to follow up all patients regularly throughout the disease course. As a result, patients are often discharged from the service following diagnosis, stabilization on any medication, and when initial care needs have been met. Some patients go on to need further and more intensive psychiatric support, including intensive care in the community through psychiatric ‘crisis teams’ or admission to specialist dementia wards in psychiatric hospitals, though the proportion of patients needing these services is not known (2,3). Clinical criteria for entry to crisis team care varies, but in Cambridgeshire patients are considered appropriate if their clinical condition is such that without intensive support they would be at significant risk of harm or require admission. Patients are considered for inpatient care if it is not possible to provide appropriate or safe care in the community.

Given this context, attempts have been made to identify risk factors for several healthcare outcomes among patients with dementia. Knapp *et al*. 2016 examined the records of 3,000 patients with Alzheimer’s disease and identified a number of demographic and clinical characteristics associated with increased risk of nursing home, general hospital, or psychiatric hospital admission (4). They analysed characteristics of patients relatively close to the point of needing increased care when differences may be maximized and the time for effective intervention may be limited. Similarly, Sommerlad *et al*. 2019 found high rates of admission to general hospitals in patients with dementia (>50% in the first year after diagnosis) and described risk factors for admission including comorbid psychiatric disorder and socioeconomic deprivation (5). The previous focus on admission to general (acute) hospitals is understandable, but the reasons for admission are likely to include physical health problems common in older patients (such as infections or fractures) which might not be due to dementia *per se* and may not be as amenable to intervention from psychiatric services. We are not aware of any studies which have examined specifically the association between factors measurable at the point of diagnosis and later need for psychiatric hospital admission. Furthermore, the number of psychiatric beds has declined in the United Kingdom and been replaced in part with intensive community support from crisis teams (6). We are not aware of any studies which have looked at risk factors at the point of diagnosis which predict need for intervention by these teams.

In this study, we examined the differences between patients with dementia who later needed inpatient psychiatric care or crisis team intervention, versus those who did not, at the point of diagnosis. If patients at high risk of needing these interventions can be reliably identified, this brings the possibility of trialling an intervention targeted to those at highest risk.

Our objectives were: (1) to establish the proportion of patients diagnosed with dementia who subsequently require psychiatric admission or intensive community support; (2) to establish the mean time between first contact with dementia services and the need for enhanced support; (3) to compare clinical information collected routinely at the point of diagnosis between patients who went on to need enhanced care and those who did not; (4) to use any such differences to explore the feasibility of developing mathematical risk prediction models suitable for defining a high-risk cohort, in support of future interventional studies; and (5) to see if such an approach could be replicated in an independent dataset.

## Methods

Secondary mental health care services in Cambridgeshire and Peterborough are provided by a single National Health Service (NHS) Trust, Cambridgeshire and Peterborough NHS Foundation Trust (CPFT), which covers the combined county and some neighbouring areas to provide a total catchment population of ~0.89 million people, of whom approximately 157,000 people are over 65 years old (7). CPFT’s memory assessment service provides between 2,000 and 2,500 assessments every year, and there are estimated to be 8,600 people with dementia in Cambridgeshire (8).

### Source data and subjects

The electronic patient records analysed for our original analysis were obtained from CPFT’s RiO clinical records system, designed by CSE Servelec, and operational 2013–2020 for older people’s mental health services in CPFT. Records were de-identified into the CPFT Research Database. We examined patients with a diagnosis of dementia, judged by the presence of clinician-coded World Health Organization (WHO) International Classification of Diseases (Tenth Edition) (ICD-10) codes starting F00 to F03, G3, and F06. We excluded those diagnosed with F06 (mild cognitive impairment (MCI)) unless they subsequently received a diagnosis of dementia. All patients examined had a diagnosis recorded between 2013 and 2020 inclusive.

**Figure 1:**
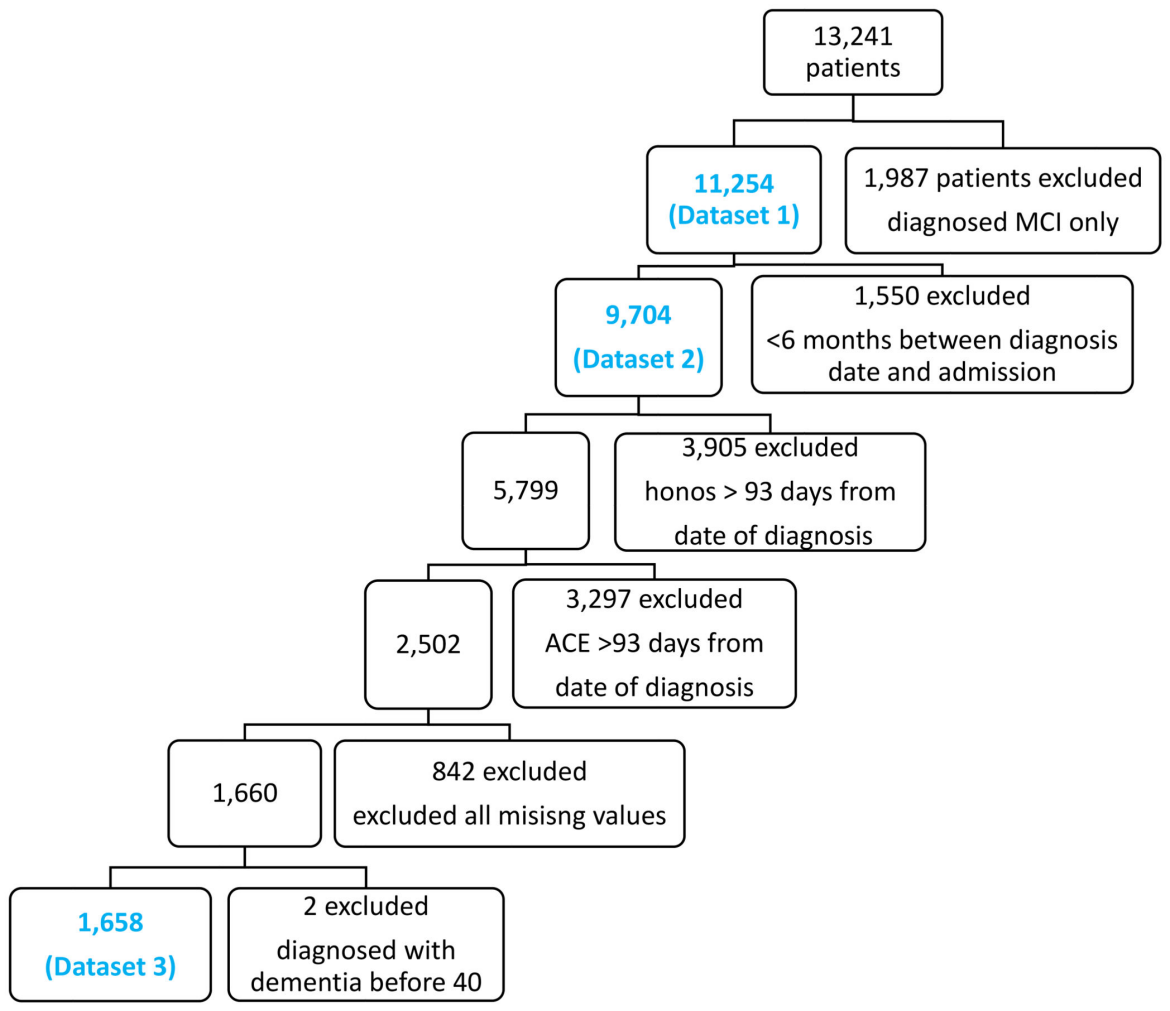
Patient population for three CPFT Datasets Primary CPFT Patient Sample. 13,241 patients in the original data set with both MCI and dementia. After excluding those with MCI only, 11,254 patients had dementia (dataset 1). 9, 704 patients with dementia had greater than 6 months between their first diagnosis and admission to crisis or inpatient (dataset 2). Out of those patients, 2,502 had a HoNOS (Health of the Nation Outcome Scale) and ACE (Addenbrooke’s Cognitive Examination) score within 93 days of diagnosis date. Unknown values excluded were missing ace, deprivation index, gender, marital status, and ethnicity. After excluding all unknown values and those diagnosed before the age of 40, there were 1,658 patients with a clean dataset (dataset 3). Abbreviations: mild cognitive impairment (MCI).

Our primary outcome was the future need for intensive community (crisis team) support or inpatient psychiatric admission, collectively referred to here as enhanced support or care. We analysed three cohorts of patients in our initial analysis (Figure 1). First, we analysed all patients with a diagnosis of dementia (dataset 1). Second, we analysed those with this diagnosis but excluding those who had less than six months between the diagnosis of dementia and needing intensive support (dataset 2), to avoid analysing patients who were diagnosed at a point of crisis (which might maximise differences between patient groups, and at a time point less amenable to intervention). Third, we examined those for whom a complete set of variables was available (dataset 3).

### Variables examined

- Date of referral to secondary care mental health services and date of diagnosis. The date of diagnosis of dementia was used as a reference point throughout the study.
- Date of death was compared for the two groups (those later requiring intensive support and those not), to allow for differential death rates as a confounding variable.
- Cognitive test scores: Patients had results from the Addenbrooke’s Cognitive Examination (ACE III) (10), mini-Addenbrooke’s Cognitive Examination (mini-ACE) (10), Mini-Mental State Examination (MMSE) (11), or Montreal Cognitive Assessment (MoCA) (12). The ACE was used for analysis as this is the standard cognitive test used in these services and accounted for the majority of the cognitive data. When excluding patients with scores reported >93 days from diagnosis, all patients analysed in the final data set had an ACE score.
- Health of the Nation Outcome Scale (HoNOS) scores: HoNOS is a standard multi-domain clinician-rated assessment, including ratings of cognitive and behavioral function, that was given to patients routinely both during initial assessment and at discharge (9). We had HoNOS scores for patients <65 years of age and HoNOS65+ scores for those aged ≥65. The HoNOS includes 12 categories, scored 0–4 (a score of 0 indicates the problem is least serious and 4 the most). The categories are behavioural disturbance, self-harm, substance misuse, cognitive problems, physical illness/disability, hallucinations/delusions, depressed mood, other mental/behavioural problems, relationship problems, activities of daily living (ADL), living conditions, and occupation/activities. The disability sub-score includes physical problems such as those due to hearing and vision impairment, medication side effects, or other injuries (13). The 12 category scores for each patient were summed to determine their total score, with 48 being the maximum.
- Index of Multiple Deprivation: This national deprivation index, derived from postcode of residence, measures seven categories including income, employment, education, health, crime, barriers to housing and services, and living environment. There are 32,844 neighbourhoods in England, and the index is a rank: 1 indicates the most deprived area and 32,844 the least deprived (14). The deprivation index was split into five quintiles such that patients in quintile 1 were from the most deprived areas and those in quintile 5 were in the least deprived.
- Marital status: Patient marital status was listed in the database as civil partnership, cohabiting, married, separated, divorced, single, not known. Patients with unknown marital status were excluded from the analysis.
- Ethnicity: The ethnicity national codes were listed in the electronic record. Any patients with unknown ethnicity were excluded.
- Age: The age at patient’s diagnosis was recorded in the database.
- Sex: The patients’ biological sex was reported as male or female.
- Diagnostic codes: All patients examined had a diagnosis of dementia. The codes for dementia included: Alzheimer’s dementia (F00/G30), vascular dementia (F01), dementia in other diseases (F02), unspecified dementia (F03). Some patients also had additional diagnoses recorded which included delirium (F05), mild cognitive impairment (F06.7, though only those patients who also had a subsequent diagnosis of dementia were included), substance use (F1), severe mental illness (F2/F30/F31), depression (F32/F33), anxiety (F40/F41), obsessive–compulsive disorder (OCD) (F42), and stress/adjustment reactions (F43).

### Statistical analysis

Due to non-normal distributions of the data, continuous variables were analysed using the nonparametric Mann–Whitney U test. For the categorical variables marital status, and ethnicity, a chi-square test of contingency was first performed. The contingency was between (a) group (patients who went on to require crisis/inpatient services versus those who did not), and (b) marital status, and ethnicity. Individual Fisher’s exact tests were performed on each of the categories within these groups. To correct for multiple comparisons, *p* values were adjusted using the Holm method (15). Fisher’s exact test was also performed for the categorical variable gender. R version 4.0.3 was used for the analysis (16). We completed a post-hoc analysis of some other variables in response to reviewers’ comments, for example use of cholinesterase inhibitors or memantine, but these were not significantly different between the groups and did not enhance the accuracy of the model. Data on patient’s place of residence or care home was not available at patient’s first diagnosis date.

We predicted the requirement for crisis/inpatient care (“enhanced support or care”). The binary outcomes examined were whether patients did or did not require a crisis team or admission to an inpatient unit within 1 year, 2 years, 3 years, etc. of their diagnosis. The follow-up time for the study was 10 years. Follow-up for each patient was until they were admitted to crisis or inpatient care, died, or the study ended (whichever occurred first). Predictors were the following: age, ACE score, HoNOS subscores, gender, ethnicity, marital status, diagnostic codes, and deprivation index.

We examined eight different models and compared their ability to predict the number of patients needing enhanced support based on the area under the receiver operating characteristic curve (AUROC) values of each model for years 1- 4 from patient’s diagnosis date. For example, the binary outcome for the model at year 2 examined whether patients were or were not admitted to high-risk units within 0-2 years inclusive. Models built included linear discriminant analysis (LDA), logistic regression (via a generalized linear model, GLM), classification and regression trees (CART), k-nearest neighbors (KNN), neural network (NN), naïve Bayes (NB), support vector machine (SVM), and random forest (RF). The models were trained and tested by using 80% of the data for the training data set and 20% for the test data set. Cross-validation was performed five times for each model. Where models were tied (for example LDA and GLM yielded similar results, as we might expect given that they are closely related), we favoured the simpler and most directly explicable (i.e. GLM in this case). AUROCS generated by LDA and GLM from the data were, as expected, very similar (at 1 year: 0.78 for both models for dataset 1 and 0.75 for dataset 2).

As logistic regression (GLM) was the optimal model (see Results), the *t* statistic was used to rank the predictors in order of importance (specifically, by |*t*|). The *car* package in R was used to assess for multicollinearity. Logistic regression assumes that there are not perfect correlations between the predictors (17). We examined the variance inflation factor (VIF), which indicates if coefficients are increased due to correlations with other predictors. The standard threshold of two was chosen (17). For datasets 1 and 2, only 2 predictors had a VIF > 2 and when these were excluded the AUROC of the model did not change significantly. (With the GLM, for dataset 1 the AUC changed to 0.74 to 0.79 and for dataset 2 the AUC changed from to 0.7 to 0.76.)

After development of the model, we examined absolute and relative risks for needing enhanced support among strata with the highest predicted risk.

### Missing values and sensitivity analysis

Many patients in the primary sample did not have a complete data set at the time of diagnosis. Not all patients had a cognitive test score, ethnicity, marital status, deprivation index, and gender reported in the electronic record. When datasets 1 and 2 were analysed, an assumption was made that the values were missing at random. In a sensitivity analysis, we used the R package, MICE (Multiple Imputations by Chained Equations), to predict missing values.

### Replication

Replication was conducted using de-identified patient records from the South London and Maudsley NHS Foundation Trust (SLaM) (18). Data from SLaM was ascertained via the Clinical Record Interactive Search (CRIS) resource. SLaM serves a local population of 1.36 million residents in the ethnically and socially diverse South London Boroughs of Croydon, Lambeth, Lewisham, and Southwark. CRIS is the anonymised version of SLaM’s record system ‘Electronic Patient Journey’. It provides research access to more than 500,000 health records within a robust governance framework (18,19). In line with the whole of London, the SLaM catchment area has a lower proportion of older adults (>60 years) than England (SLaM catchment: 13%; England: 22.3%) (18). Dementia diagnoses are made in memory services, and patients are only followed up beyond 3 months after the diagnosis of dementia if social or non-cognitive problems arise, a similar model to Cambridgeshire/Peterborough.

This dataset included 14,072 patients; 323 patients had a period of less than 6 months between diagnosis and enhanced care and were excluded. After excluding those with a HoNOS score and MMSE recorded more than 93 days after diagnosis, 6,729 patients were in the final SLaM dataset (Supplementary Figure 1). Of these, 2.4% received enhanced care (either inpatient admission or crisis team community support). Replication was performed by fitting a logistic regression using the same predictors in the new data set. In the replication version, HoNOS scores in each category were dichotomized (score 0–1 = absent, score 2–4 = present). We found there were some differences in the data sets which precluded full direct replication. For example, MMSE was much more commonly used in SLaM than the ACE. Though replication using exactly the same model and variables as in CPFT yielded similar results, for the main analysis of the SLaM data we used their most commonly used cognitive tool, and again found similar results. This suggests the findings are a genuine reflection of cognition rather than specific to the cognitive test used and that these results may be widely applicable, for example to other Trusts using the MMSE rather than the ACE.

### Ethics

The CPFT Research Database holds NHS Research Ethics approvals (12/EE/0407, 17/EE/0442, 22/EE/0264). The CPFT Research Database Oversight Committee further approved the project. CRIS has received ethical approval as an anonymised data resource (Oxford Research Ethics Committee C, reference 18/SC/0372).

### CPFT Data and Research Availability Statement

Raw de-identified data from the CPFT Research Database are not publicly available, under the terms of NHS Research Ethics approvals; for details of access and conditions, contact research.database@cpft.nhs.uk

### CPFT Analytic Code Availability

Code can be made available upon request from by contacting: Sabina.London@pennmedicine.upenn.edu

### SLaM Data and Research Availability Statement

All relevant aggregate data are found within the paper. The data used in this work have been obtained from the Clinical Record Interactive Search (CRIS), a system that has been developed for use within the NIHR Mental Health Biomedical Research Centre (BRC) at the South London and Maudsley NHS Foundation Trust (SLaM). It provides authorised researchers with regulated access to anonymised information extracted from SLaM's electronic clinical records system. Individual-level data are restricted in accordance to the strict patient led governance established at South London and The Maudsley NHS Foundation Trust, and by NHS Digital for the case of linked data. Data are available for researchers who meet the criteria for access to this restricted data: (1) SLaM employees or (2) those having an honorary contract or letter of access from the trust. For further details, and to obtain an honorary research contract or letter of access, contact the CRIS Administrator at cris.administrator@kcl.ac.uk.

## Results

### Subject characteristics: primary sample

The patient groups we analysed from CPFT data are shown in Figure 1 (all patients with dementia in dataset 1, those with partial data excluding those with <6 months between diagnosis and enhanced care in dataset 2, and patients with full data and excluding those less than 6 months between diagnosis and need for enhanced care in dataset 3). Characteristics of CPFT patients from the datasets 1 and 2 are summarised in Supplementary Table 1.

### Differences at the time of diagnosis between groups later needing intensive care or not

Scores on the HoNOS subcategories were significantly higher in patients who subsequently required crisis or inpatient care (‘high needs’ group) compared to those who did not in our original data set. Table 1 summarizes all variables analysed, with univariate tests for dataset 1 (summarised in Supplementary Table 2 for dataset 2).

Age, gender, marital status, and ethnicity differed significantly between groups. Patients that went on to require enhanced care were more likely to be younger and married. Enhanced community care was more common in Cambridgeshire (~3 times more common than inpatient admission). The risk factors for needing admission or enhanced community care were the same for both groups. All the variables examined were then used in the creation of a model to predict which patients were at most risk of needing crisis or inpatient care.

### Predictors of later need for crisis/inpatient care

Out of 8 models examined for dataset 1, logistic regression (a GLM) was used for the remainder of the analysis, which had AUROC values of 0.74 to 0.78 for periods of 1–4 years after diagnosis. The best alternative models performed worse than or similar to the logistic regression (see Supplementary Figure 2 for details. The AUROC was 0.71-0.75 for the dataset 2 (Supplementary Figure 3) and 0.6 to 0.65 for the 1,658 set (Supplementary Figure 4).

The odds ratio (or the arithmetic change in log odds) that the person is admitted to crisis or inpatient care and outputs of the winning model are shown in Table 2 (shown in Supplementary Table 3 for (dataset 2). Later need for intensive support was positively and significantly associated with male sex and greater behavioural disturbance, but was inversely associated with age, and the level of physical/functional impairment (judged via physical disability on HoNOS). Greater cognitive problems, as judged via the HoNOS, predicted more future need in the combined logistic regression model (which accounts for all variables simultaneously); and, similarly, worse cognition (as judged by lower ACE scores) predicted greater future need.

We considered whether death may have been a confounding variable. However, there were not significant differences in the rates of death between the two groups. For dataset 1, the proportions of those who died were 55.8% and 56.1% for those requiring and those not requiring enhanced support, respectively (X^2^=0.0418, not significant [NS]). For dataset 2, the proportions of those who died were 55.3% and 55.4% for those requiring and those not requiring enhanced support, respectively (X^2^=7.30E-05, NS). This indicates differential mortality was not a confounding variable in this case.

Supplementary Figure 5 shows receiver operating characteristic (ROC) curves for each year since the patient’s first diagnosis, from 1-4 years, for all three CPFT datasets (patients with full data, partial data, and for the whole population). All models performed well but models based on larger data sets performed better, likely due to a higher number of crisis or inpatient events in a larger population. For dataset 1, the AUROC was between 0.74 and 0.78 for the logistic model between 1-4 years since the first diagnosis date. Similarly, for 9,704 patients, The AUROC was between 0.71 and 0.75 for the logistic model.

For the complete dataset 3, the AUROC was between 0.6 and 0.65 for logistic model since the first diagnosis date. However, when replicated in London with a larger complete dataset of 6, 729 patients, the AUROC was better at 0.746 (Supplementary Figure 6).

Having established these significant predictors, we ranked them by their predictive contribution (Supplementary Figure 7). For dataset 1 and 2, the most important variables were age, dementia subtype, and behavioural scores on the HoNOS. Variables in both sets which did not significantly predict outcome were ADL, ethnicity, occupation, and self-harm.

### Identifying patients most at risk for needing crisis or inpatient care

**Figure 2:**
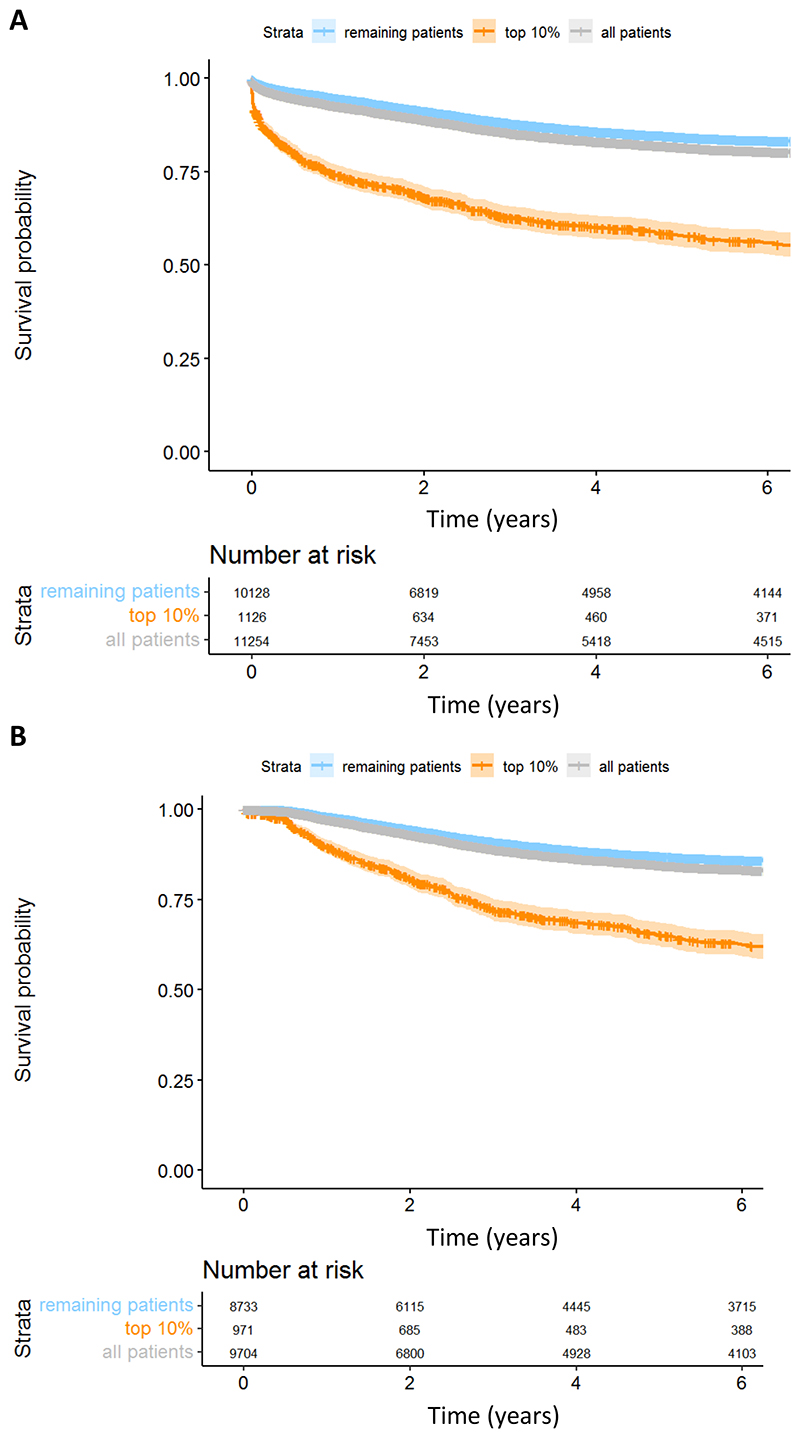
Top 10% of Patients Who are Most at Risk of Needing Enhanced Care Top ten percent of patients who are most of risk of needing crisis or inpatient care (orange line) and the remaining patients (blue line) are shown above by the Kaplan–Meier survival curve. 11,254 patients (dataset 1) are shown in Figure 2A and 9,704 patients (dataset 2) in Figure 2B. All patients in the study are shown by the grey line. Time is shown in years since the patient’s first diagnosis date. The numbers below the graph are the number of patients who have not yet required enhanced care at each timepoint.

The logistic regression model was used to determine the probabilities of patients being admitted to crisis or inpatient units over time. Based on these probabilities, the predicted top 10% of patients most at risk of needing crisis or inpatient care were identified (Figure 2). For dataset 2, those in the highest-predicted-risk cohort had an 10.6% chance of enhanced care after 1 years and a 19.3% chance after 2 years. The other 90% had a 2% chance of enhanced care after 1 years and a 5.6% chance after 2 years. Therefore, after 2 years, there was a 3.5-fold increase in enhanced care for the 10% at highest predicted risk. After 6 years, 37.5% of the highest-predicted-risk patients needed crisis or inpatient care compared to 14 % of the other 90%. When we analysed all patients (dataset 1), including those with less than 6 months between diagnosis and enhanced care (perhaps the most useful population for clinical application) we found a likelihood of needing enhanced care of 31.8% at 2 years among the 10% at highest risk, and 8.7% among the other 90% of patients.

### Subject characteristics: replication sample

The overall characteristics of the SLaM dataset are described in Supplementary Table 1. The SLaM dataset ethnic groups included 20.6% Black and 69.9% white participants. This is more ethnically diverse than the CPFT dataset which included 0.986% Black and 92.4% white participants. More of the population in SLaM was female compared to CPFT, and patients were diagnosed at an average age of 82, similar to the CPFT gender and age characteristics.

### Group differences at first contact: replication sample

Supplementary Table 4 summarizes differences between groups who later needed enhanced care, versus those who did not, using univariate analysis. Patients in the future-enhanced-care group were more likely to be younger and married, with no group differences in ethnicity or gender. Scores on four of the 12 HoNOS subcategories (behaviour, hallucinations, depression, and relationships) were significantly higher in patients who subsequently required enhanced care compared to those who did not. Scores on the disability subcategory of the HoNOS were lower for those in the future-enhanced-care group.

All the variables examined were then used in the creation of a model to predict which patients were at most risk of needing crisis or inpatient care (Supplementary Table 5).

### Logistic regression model performance in replication sample

The AUROC for the SLaM dataset/model was 0.746 (Supplementary Figure 6). The logistic regression model (accounting for multiple variables simultaneously) also found that younger patients were more likely to need crisis/inpatient care, and likewise those from more deprived areas. Although ethnicity was not a significant predictor in univariate analysis (Supplementary Table 4), in the full logistic regression model, patients who were Asian were less likely to require enhanced care (Supplementary Table 5). Higher HoNOS subscores for behavioural disturbance, depression, and relationship problems, and a lower score on the physical illness/disability subscore, were predictive of need for enhanced care.

## Discussion

In this paper we present the largest data set to date examining patients diagnosed with dementia and their subsequent risk of needing psychiatric inpatient or community crisis (“enhanced”) care. At the point of diagnosis, patients who subsequently needed enhanced care in the CPFT dataset were younger, more likely to be male, had more impaired cognition as measured by formal cognitive testing, and higher HoNOS scores, in particular for the behavioural disturbance sub-scale. We were able to use these differences to model risk with a clinically acceptable level of accuracy (AUROC 0.78–0.74) and define the 10% of patients at highest predicted risk. The process was replicated and a similar model created using the SLaM dataset which had an AUROC of 0.74. This was a more diverse population with 69.9% white and 20.6% Black patients. We were reassured overall by the lack of difference based on ethnic origin across the two databases as this is in contrast to other areas of psychiatry where access to services does show ethnic disparity (20). Consistent with the CPFT dataset, patients who needed enhanced care were younger and had higher HoNOS subscores for behavioural disturbance, hallucinations, relationships, and lower subscores for physical illness/disability. Data from both Trusts show that those from more deprived areas more often required enhanced care. Some differences were found with demographic variables and HoNOS subscores. In addition, the SLaM dataset showed that higher scores on the depression scale were predictive of those needing enhanced care.

Six years after diagnosis, only a minority of patients in Cambridgeshire needed enhanced care in the absence of routine follow-up (16% in Cambridgeshire for dataset 1, 12.8% in Cambridgeshire for dataset 2, and 12% for dataset 3). This was also true in London where only 2.4% of the total group had needed enhanced care. It may not therefore be fruitful to continue to see all patients with a dementia diagnosis with the aim of averting a deterioration requiring enhanced care, as only a minority will need it. There may be value in attempting to identify the patients who are at highest risk of needing enhanced care in order to target interventions. We identified factors measured at the time of original diagnosis that predicted which patients would later need enhanced care, even though, on average, the time to needing enhanced care for dataset 2 was 2.65 years for those requiring crisis or inpatient admission. Though our initial analysis excluded patients who needed enhanced care soon after diagnosis (within six months), so that we were predicting future rather than nascent current crises, the length of time between diagnosis and the need for enhanced care means that interventions could be implemented to attempt to improve outcomes. Subsequent analysis of all patients, including those with less than 6 months between diagnosis and enhanced care, still found an overall mean time of 1.88 years between diagnosis and the need for enhanced care, where it was needed. Interventions could be implemented and some that have been reported to bring benefit include: “case management” has reduced long-term placement (e.g. residential care placement), though with a lesser or no impact on hospital admission (21). Targeting such interventions more specifically at those at highest risk, and for outcomes where they might be more successful, such as crisis events, might yield better results.

Clinical services supporting patients with dementia in the UK could feasibly focus on those most at risk, as the number of patients in this group is not prohibitively large. For example, the memory assessment services in Cambridgeshire routinely see ~2,500 patients a year. Resource limitations mean not all patients can be supported by secondary care services indefinitely, but focusing on the 10% most at risk would mean following up 250 patients per year. Based on the present analysis for dataset 1, after 2 years, focusing resource on the 250 patients most at risk would cover approximately 78 patients who would have needed enhanced care. Importantly the parameters included here, including those used for modelling, were derived from data already collected in routine clinical practice: No extra time would be required from the clinical team to record data to identify patients at highest risk of needing enhanced care. Furthermore, we were able to identify similar factors predicting need for enhanced care in a second Trust with very different patient demographics, differences in the data they record, and differences in service provision, and use these to build models with similar levels of accuracy. This suggests such an approach may be widely applicable.

Our study has some limitations. As a retrospective cohort study, it is at risk of including confounding variables. The time period examined here also included a global pandemic, which impacted rates of referral, diagnosis and inpatient admission (22). Though all services were disrupted to some extent, crisis and inpatient services continued to function throughout the pandemic. Our cognitive data in the CPFT cohort was based on ACE scores. Though this test was completed by the majority of patients in our dataset, the choice of cognitive test might be related to other clinical features which may themselves relate to risk of needing enhanced care; for example, those most cognitively impaired might complete an alternative cognitive test if they are not able to tolerate a full ACE. Our original model was run on a minority of patients in order to exclude patients with missing variables; however, subsequent analyses on the total population and an independent data set obtained similar results in terms of ability to predict need for enhanced care. We acknowledge that admission or crisis team intervention is only one (proxy) outcome measure, and further work should explore other possibilities – for example, quality of life measures, or time to institutional living. We found some differences between the two Trusts, for example in the proportion of patients receiving inpatient or crisis care. This most likely represents a difference in service provision between the two Trusts but is itself interesting and warrants further investigation. Lastly, though our approach involved independent replication in a second NHS Trust, other organisations may need to develop a tool based on their locally available data, as data sets are not uniform across the UK. Nevertheless, the similar results in two different Trusts do suggest that the identification of patients at the point of diagnosis is possible and likely to extend to different populations using only routinely collected data.

In summary, our findings suggest that only a minority of patients with dementia deteriorate to require inpatient psychiatric admission or community crisis team care over 6 years after diagnosis, and those who are at highest risk can be identified at the time of diagnosis, many months or years before they reach crisis point. This raises the possibility of intervening in a targeted fashion. Such an intervention might be as simple as continued contact with secondary care services rather than discharge to primary care, or more active interventions such as case management (21). The logical extension of our work would be an intervention trial in the high-risk cohort we have been able to identify, to see if outcomes can be improved. If successful, this would have a positive impact on healthcare systems and would likely be welcomed by patients and their carers.

## Supplementary Material

Supplementary material

## Figures and Tables

**Table 1:** Variables Examined for 11,254 Patients in CPFT Dataset Characteristics of patients needing enhanced care compared to those who did not. The percent of patients with a Health of the Nation Outcome Scale (HoNOS) subscale score of >=2 is indicated in the table as this score was taken to indicate the presence of a problem. The Addenbrooke’s Cognitive Examination (ACE) is the cognitive test score reported in the table. (Bold items are significant; *p <0.05, ** p <0.01, *** p <0.001). Three significant figures are used in the table.

Variable	Crisis or Inpatient	None	P Value
***Age Mean ± SD	79.1 ± 9.49	82.4 ± 8.19	<2.2E-16
*ACE Mean ± SD	57.9 ± 19.9	59.1 ± 19.0	0.0359
***HoNOS Mean ± SD	9.69 ± 5.60	7.01 ± 5.47	<2.2E-16
***Behavior	23.5%	9.45%	<2.2E-16
***Self-Harm	1.84%	0.677%	<2.2E-16
***Cognitive	76.4%	67.6%	<2.2E-16
**Disability	46.4%	44.3%	0.00117
***Substance Use	2.67%	1.11%	1.05E-10
***Hallucinations	17.8%	9.38%	<2.2E-16
***Depressed mood	19.7%	11.7%	<2.2E-16
***Other Mental	36.1%	19.6%	<2.2E-16
** Behavioral Problems**			
***Relationships	19.5%	8.95%	<2.2E-16
***Living Conditions	7.46%	4.46%	4.04E-13
***ADL	53.4%	46.5%	<2.2E-16
***Occupation	19.9%	15.1%	8.57E-14
***Gender			7.20E-13
Female	51.1%	60.3%	
Male	48.9%	39.7%	
***Marital Status			<2.2E-16
Married	51.8%	38.7%	
Not Married	48.2%	61.3%	
*Ethnicity			0.0137
**White	93.9%	92.1%	0.00875
Asian	1.67%	1.65%	0.920
Black	0.501%	1.08%	0.0191
Other	3.95%	5.17%	0.0285

**Table 2:** Output of the Logistic Regression for 11,254 Patients in CPFT Dataset Output of the logistic regression. All the variables used in the model are shown above. For ethnicity, white was used as the reference. For deprivation index, IMD 2, IMD 3, IMD 4, IMD least deprived are the 2^nd^, 3^rd^, 4^th^, and 5^th^ quintiles, respectively. IMD 1 was used as the reference. Bold items are significant; *p <0.05, ** p <0.01, *** p <0.001. Three significant figures are used in the table.

Variable	Odds Ratio	Std Error	Z	*p*
***(Intercept)	3.279	0.308	3.853	0.000117
***Age at Diagnosis	0.968	0.003	-10.121	< 2e-16
***Gender: Male	1.386	0.057	5.731	9.98E-09
***Married	1.322	0.057	4.874	1.10E-06
Ethnicity				
*Ethnic: Black	0.447	0.361	-2.228	0.026
Ethnic: Asian	0.817	0.213	-0.949	0.343
*Ethnic: Other	0.755	0.136	-2.065	0.039
Deprivation				
*Deprivation: IMD2	0.817	0.086	-2.36	0.018
Deprivation: IMD3	0.895	0.084	-1.318	0.188
*Deprivation: IMD4	0.829	0.085	-2.207	0.027
**Deprivation: IMD5 (least deprived)	0.781	0.086	-2.852	0.004
Diagnosis Codes				
***Dementia Alzheimer’s	0.556	0.073	-8.049	8.33E-16
***Dementia Vascular	0.377	0.104	-9.398	< 2e-16
***Dementia Unspecified	0.443	0.118	-6.893	5.45E-12
***Dementia Other	0.343	0.148	-7.208	5.67E-13
HoNOS				
***Behavioural Disturbance	1.342	0.038	7.803	6.03E-15
Self Harm	1.064	0.08	0.781	0.435
*Substance Use	1.204	0.072	2.589	0.01
***Cognitive	1.188	0.036	4.861	1.17E-06
***Disability	0.891	0.03	-3.897	9.73E-05
***Hallucinations	1.194	0.034	5.229	1.70E-07
Depressed Mood	1.046	0.037	1.213	0.225
***Other	1.179	0.029	5.635	1.75E-08
***Relationships	1.152	0.038	3.724	0.000196
ADL	0.968	0.034	-0.952	0.341
Living Conditions	1.091	0.047	1.84	0.066
Occupation	0.966	0.037	-0.932	0.352
***ACE	0.994	0.001	-3.713	0.000205

## Research in context

### 1 Evidence before this study

Support after diagnosis is a major concern to patients with dementia and their carers. Universal provision of such support throughout the course of the condition is challenging, so targeting interventions may be attractive if patients at highest risk of deterioration and subsequent need for enhanced care can be identified at the time of diagnosis. Previous work has identified risk factors and predictive models for patients with dementia and admission to general hospitals. Much less work has been done on risk factors for admission to psychiatric hospitals, particularly for predicting risk at time points substantially before admission, and none, as far as we are aware, to identify risk factors for needing intensive community (crisis team) support.

### 2 Added value of this study

This study addresses a gap in the literature and demonstrates that patients with dementia who go on to need inpatient psychiatric or intensive community care can already be differentiated from those who do not at the point of diagnosis using routinely collected clinical data. These data can be used to create models which identify those patients at highest risk.

### 3 Implications of all the available evidence

The ability to identify high-risk patients allows for trials of targeted clinical interventions to improve outcomes.
