# Supplementary material for: Predicting patients with dementia most at risk of needing psychiatric inpatient or enhanced community care using routinely collected clinical data: a retrospective multi-site cohort study
Source: Br J Psychiatry. Author manuscript; Available in PMC 2024 Jun 1. (PMC7615978; doi:10.1192/bjp.2024.14)
Supplement: Supplementary material [file EMS193254-supplement-Supplementary_material.pdf]

Supplementary Figure 1: Characteristics of Overall Patient Population in SLaM Data

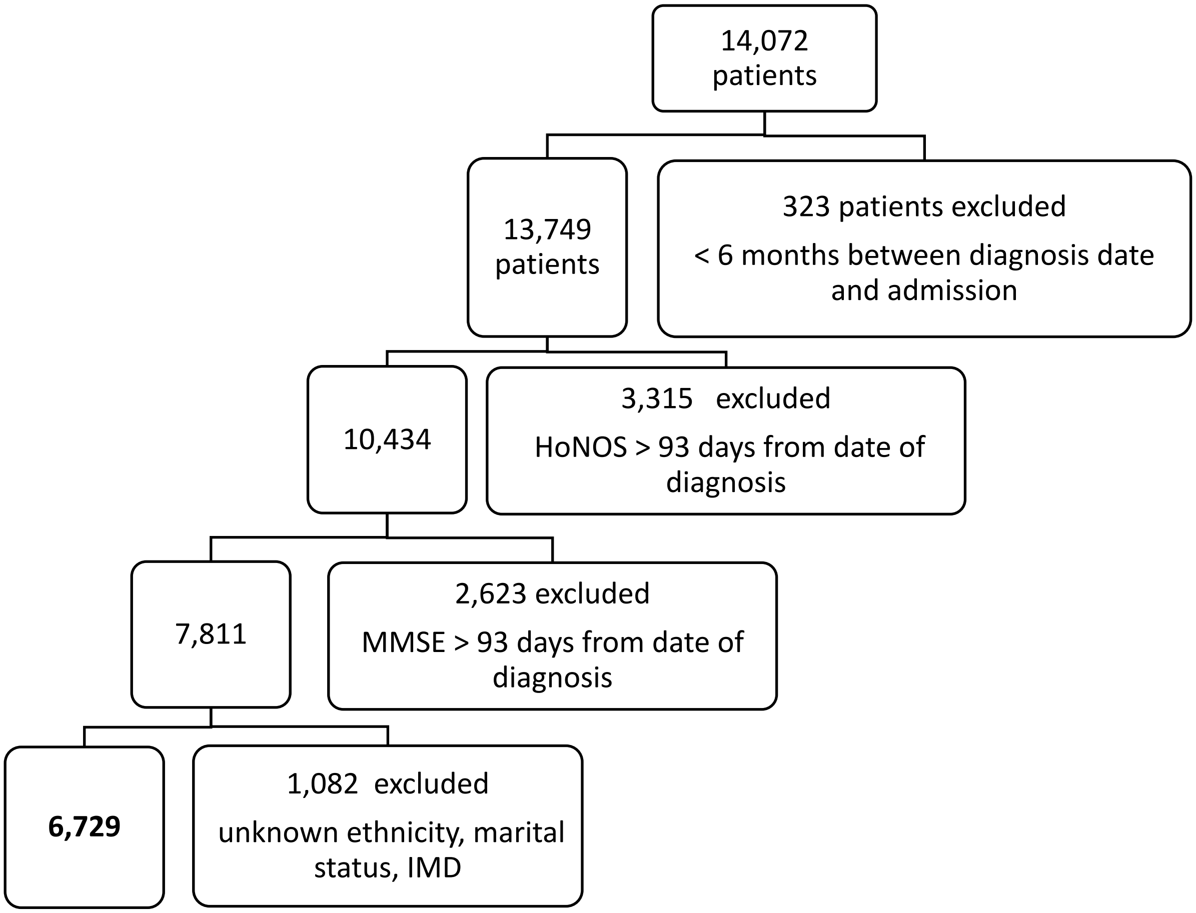

Supplementary Figure 1: Patient Population. 14,072 patients in the original data set. After the exclusion criteria, there were 6,729 patients with a full data set analysed for the study. Abbreviations: Health of the Nation Outcome Scale (HoNOS), Index of Multiple Deprivation, Mini-Mental State Examination (MMSE), Index of Multiple Deprivation (IMD).

Supplementary Table 1: Characteristics of Patients Included in in CPFT and SLaM Datasets

Variable	CPFT (n=11,254)	CPFT (n=9,704)	SLaM (n=6,729)
Sociodemographic Variables			
Age at Diagnosis (mean, SD)	81.9 (8.50)	82.0 (8.38)	82.1 (7.7)
Female (%)	58.8%	59.5%	60.8%
Ethnicity			
White (%)	92.4%	92.4%	69.9%
Black (%)	0.986%	1.04%	20.6%
Asian (%)	1.65%	1.65%	6.30%
Other (%)	4.98%	4.96%	3.20%
Married or Cohabiting (%)	40.8%	40.3%	32.8%
ACE / MMSE score (mean, SD)	58.9 (19.1)	59.2 (18.9)	17.4 (6.7)
Total HoNOS score[1]	7.44 (5.58)	6.89 (5.26)	10.2 (5.30)
Mental Health Problems according to HoNOS[1]			
Behaviour Disturbance (%)	11.7%	8.34%	15.4%
Self Harm (%)	0.862%	0.484%	1.20%
Substance Use (%)	1.36%	1.21%	3.10%
Cognitive Problems (%)	69.1%	69.0%	84.8%
Hallucinations (%)	10.7%	8.38%	10.9%
Depressed Mood (%)	12.9%	11.0%	13.7%
Physical illness or disability according to HoNOS[1]	44.6%	43.8%	54.2%
Functional problems according to HoNOS[1]			
Relationships (%)	10.6%	8.06%	12.6%
ADL (%)	47.6%	45.9%	57.9%
Living Conditions (%)	4.94%	4.06%	11.6%
Occupation (%)	15.8%	15.0%	31.9%

Supplementary Table 1: Characteristics of patients included in final analysis in CPFT and SLaM. Three significant figures were used in the table. Values shown are mean (SD) or percentage. [1]The percent of patients with a Health of the Nation Outcome Scale (HoNOS) subscale score of >=2 is indicated in the table as this score was taken to indicate the presence of a problem.

Supplementary Table 2: Baseline Characteristics of Patients Requiring Enhanced Care Vs Not in CPFT Dataset (9,704 patients, dataset 2)

Variable	Crisis or Inpatient (n = 1,246)	None (n = 8,458)	P Value
*****Age at Diagnosis**	78.6 ± 9.66	82.5 ± 8.05	<2.2E-16
ACE	58.5 ± 19.5	59.3 ± 18.6	0.206
*****HoNOS Total**	8.97 ± 5.20	6.59 ± 5.19	<2.2E-16
*****Behavioural Disturbance**	18.4%	6.86%	< 2.2E-16
*****Self Harm**	1.12%	0.390%	1.42E-09
*****Cognitive**	78.2%	67.7%	< 2.2E-16
*****Disability**	44.1%	43.8%	0.109
*****Substance Use**	2.65%	0.993%	7.02E-07
*****Hallucinations**	14.0%	7.55%	< 2.2E-16
*****Depressed Mood**	16.9%	10.1%	< 2.2E-16
*****Other Mental/Behavioural ***Problems**	31.9%	16.9%	< 2.2E-16
*****Relationships**	15.7%	6.94%	< 2.2E-16
***** Living Conditions**	6.18%	3.75%	1.97E-07
*****ADL**	50.4%	45.3%	4.23E-11
*****Occupation**	19.5%	14.35%	3.70E-12
*****Gender**			1.55E-09
Female	51.6%	60.7%	
Male	48.4%	39.3%	
*****Marital Status**			< 2.2e-16
Married	54.4%	38.2%	
Not married	45.6%	61.8%	
****Ethnicity**			0.00986
****White**	94.5%	92.0%	0.00137
Asian	1.36%	1.69%	0.475
Black	0.482%	1.12%	0.0356
Other	3.61%	5.15%	0.0174

Supplementary Table 1: Characteristics of patients later in need of crisis/inpatient admission compared to those who did not later need this. Age, ACE, HoNOS total and 12 subcategories (behavioural disturbance, self-harm, cognitive, disability, substance use, hallucinations, depressed, other mental/behavioural problems, relationships, living conditions, ADL, occupation) are shown. Other variables analysed included gender, marital status, ethnicity, and diagnosis codes. Bold items are significant; * p<0.05, ** p<0.01, *** p<0.001. Three significant figures are used in the table.

Supplementary Figure 2: Area Under the Receiver Operating Characteristic (ROC) Curves for 8 Different Models in CPFT (11,254 Patients)

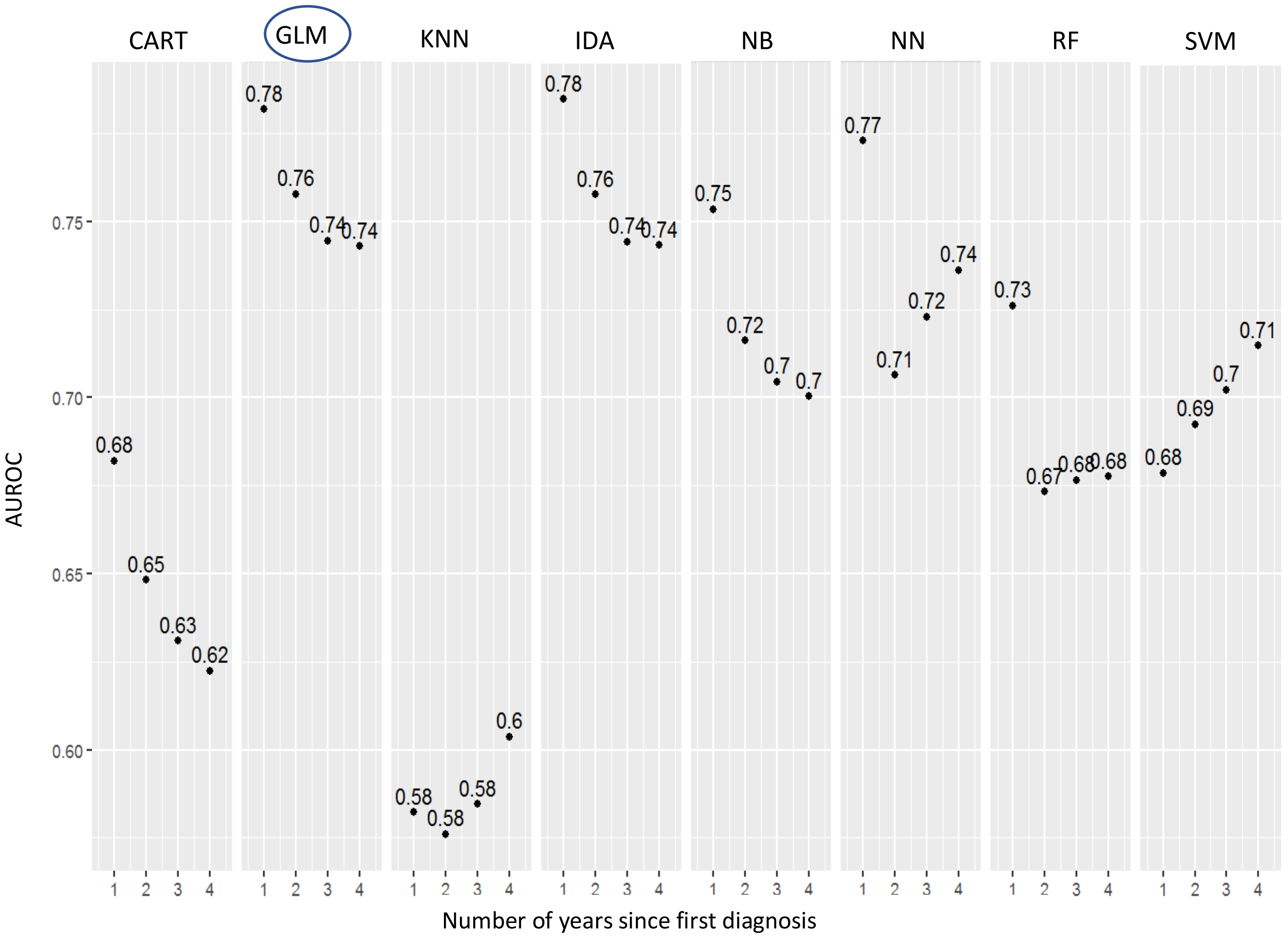

Supplementary Figure 2: Area under the receiver operating characteristic curve (AUROC) calculated for 8 different models examined including linear discriminant analysis (LDA), generalized linear model or logistic regression (GLM), decision tree (CART), k-nearest neighbors (KNN), neural network (NN), naïve Bayes (NB), support vector machines (SVM), and random forest (RF). The models were trained and tested by inputting 80% of the data into the training data set and 20% into the test data set. The AUROC was between 0.74 and 0.78 for GLM and LDA between 1-4 years after diagnosis. The linear discriminant analysis functions by taking the existing data from the model and projecting it onto a new dimensional space (Linear Discriminant Analysis, 2022). Although both GLM and LDA models had high AUROC values, GLM was chosen to determine the top 10% of patients needing intensive care since this is a simple and readily explicable model. The K-nearest neighbors algorithm, neural network, decision tree, and support vector machines had lower AUROC values ranging from 0.58 to 0.77. Overall, almost all the models had high predictive ability except for the K-nearest neighbors model.

Supplementary Figure 3: Area Under the Receiver Operating Characteristic (ROC) Curves for 8 Different Models in CPFT (9,704 Patients)

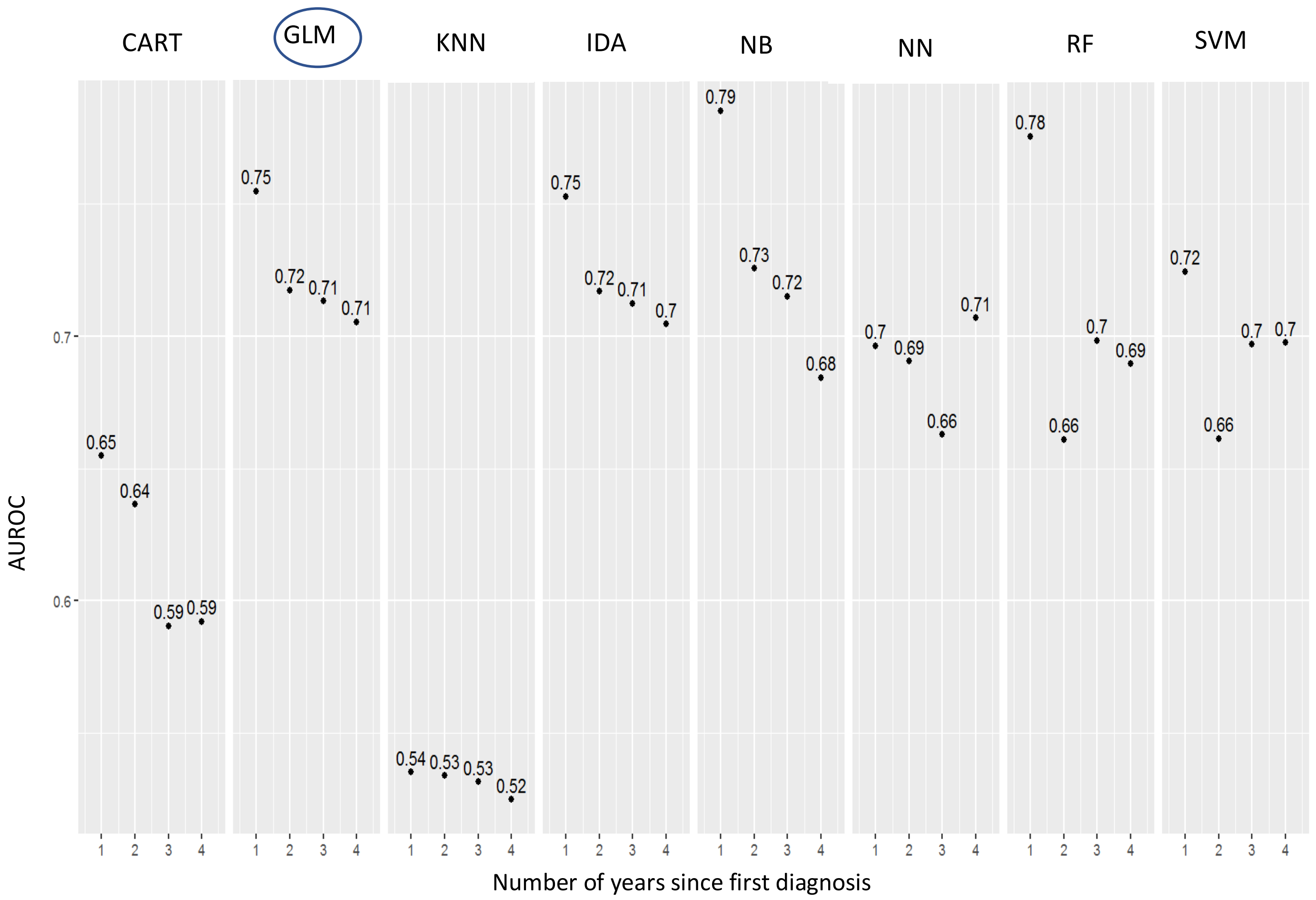

Supplementary Figure 3: Area under the receiver operating characteristic curve (AUROC) calculated for 8 different models as in Supplementary Figure 2. The AUROC was between 0.71 and 0.75 for GLM and 0.7 to 0.75 for LDA between 1-4 years after diagnosis. Although both GLM and LDA models had high AUROC values, GLM was chosen to determine the top 10% of patients needing intensive care since this is a simple and readily explicable model. The K-nearest neighbors algorithm, neural network, decision tree, and support vector machines had lower AUROC values ranging from 0.59 to 0.72. Overall, almost all the models had high predictive ability except for the K-nearest neighbors model.

Supplementary Figure 4: Area Under the Receiver Operating Characteristic (ROC) Curves for 8 Different Models in CPFT (1,658 Patients)

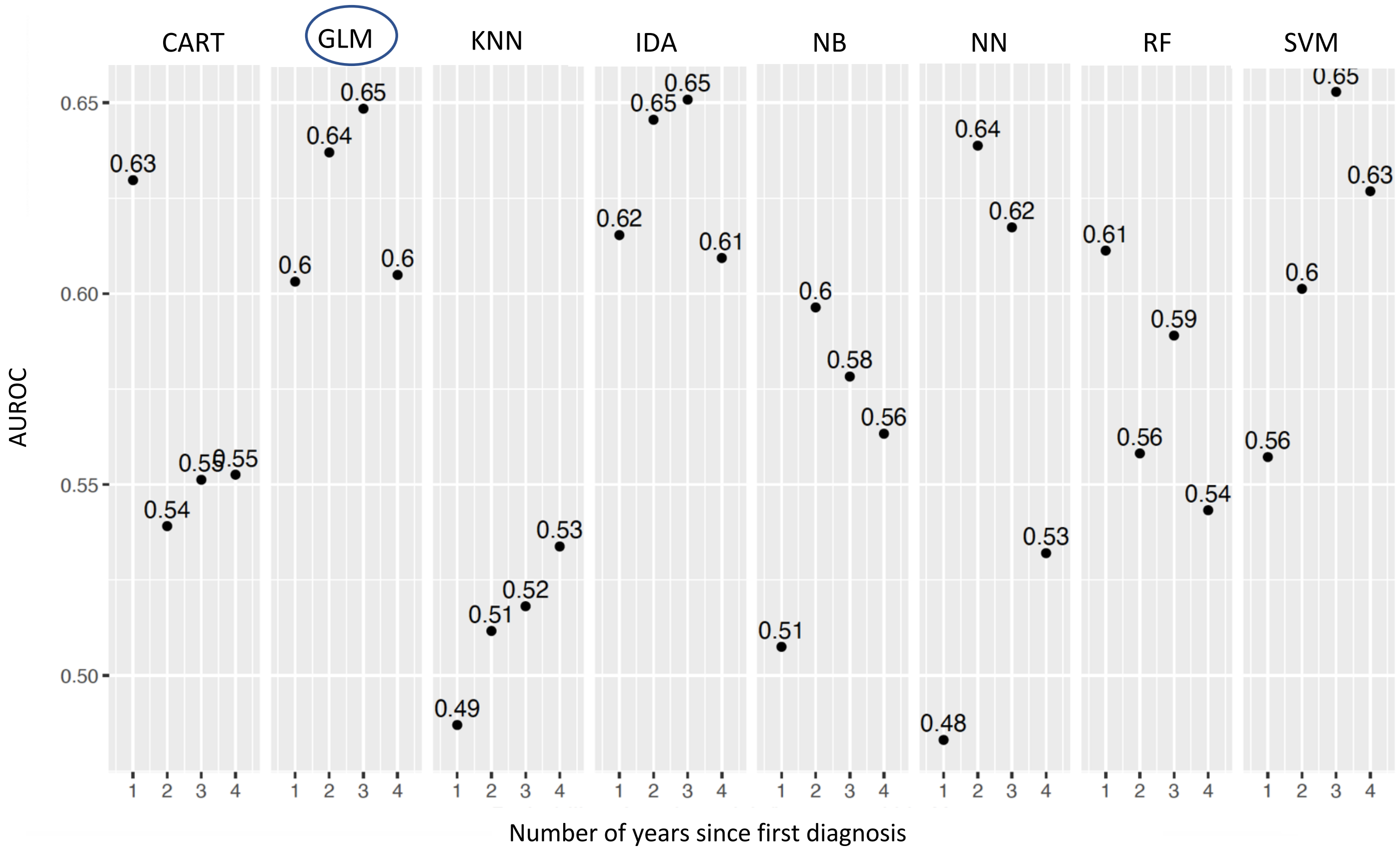

Supplementary Figure 4: Area under the receiver operating characteristic curve (AUROC) calculated for 8 different models as in Supplementary Figure 2. The AUROC was between 0.6 and 0.65 for GLM and 0.61 to 0.65 for LDA between 1-4 years after diagnosis. Although both GLM and LDA models had high AUROC values, GLM was chosen to determine the top 10% of patients needing intensive care since this is a simple and readily explicable model. The K-nearest neighbors algorithm, neural network, decision tree, and support vector machines had lower AUROC values ranging from 0.49 to 0.65. Overall, almost all the models had high predictive ability except for the K-nearest neighbors model.

Supplementary Table 3: Logistic Regression Predicting the Need for Enhanced Care in CPFT (9,704 patients, dataset 2)

Variable	Odds Ratio	Std Error	Z	p
***(Intercept)	4.078	0.362	3.88	1.04E-04
***Age at Diagnosis	0.96	0.004	-10.694	< 2E-16
***Gender: Male	1.373	0.067	4.711	2.47E-06
***Married	1.457	0.068	5.56	2.70E-08
Ethnicity				
*Ethnic: Black	0.361	0.441	-2.309	0.021
Ethnic: Asian	0.594	0.274	-1.898	0.058
*Ethnic: Other	0.652	0.168	-2.547	0.011
Derivation				
*Deprivation: IMD2	0.817	0.102	-1.988	0.047
Deprivation: IMD3	0.928	0.1	-0.748	0.454
Deprviation: IMD4	0.94	0.099	-0.625	0.532
*Deprivation: IMD5 (least deprived)	0.799	0.103	-2.179	0.029
Diagnosis Codes				
***Dementia Alzheimer's	0.639	0.086	-5.218	1.80E-07
***Dementia Vascular	0.351	0.132	-7.932	2.16E-15
***Dementia Unspecified	0.527	0.141	-4.545	5.49E-06
***Dementia Other	0.376	0.177	-5.515	3.48E-08
HoNOS				
***Behavioural Disturbance	1.376	0.048	6.646	3.00E-11
Self Harm	1.044	0.12	0.354	0.724
*Substance Use	1.25	0.089	2.509	0.012
***Cognitive	1.243	0.043	5.118	3.08E-07
***Disability	0.869	0.035	-3.968	7.26E-05
***Hallucinations	1.24	0.044	4.861	1.17E-06
Depressed	1.039	0.046	0.845	0.398
***Other	1.184	0.036	4.744	2.09E-06
***Relationships	1.186	0.048	3.577	0.000348
ADL	0.932	0.041	-1.733	0.083
Living Conditions	1.109	0.059	1.741	0.082
Occupation	0.997	0.044	-0.065	0.949
**ACE	0.995	0.002	-2.876	0.004

Supplementary Table 3: Output of the logistic regression. All the variables used in the model are shown above. For ethnicity, white ethnicity was used as the reference category. IMD are the quintiles for the Index of Multiple Deprivation. IMD1 (most deprived quintile) was used as the reference category. The reference category for diagnosis codes was ICD 10 F06 (dementia due to brain injury). Bold items are significant; *p <0.05, ** p <0.01, *** p <0.001. Three significant figures are used in the table.

Supplementary Figure 5: ROC Curves for Logistic Regression Model for 3 CPFT Datasets

A. Dataset 1

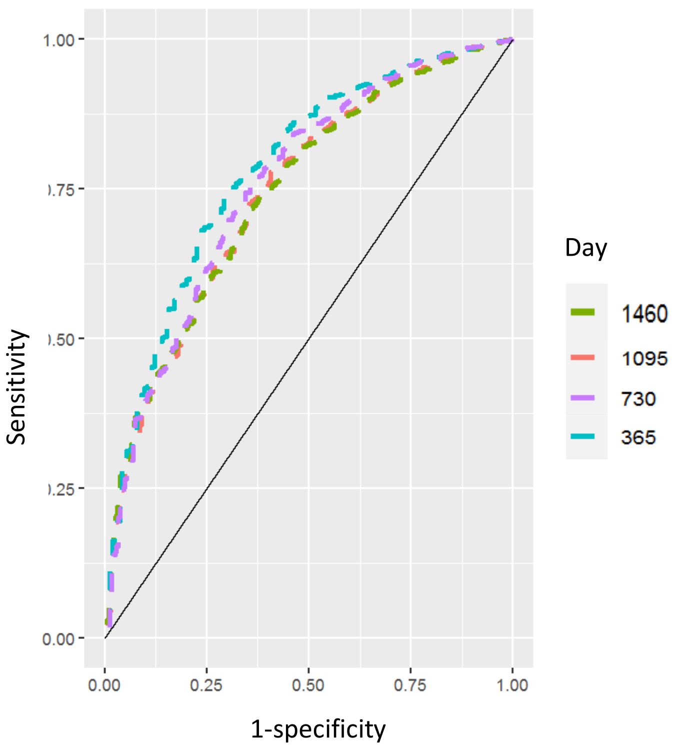

B. Dataset 2

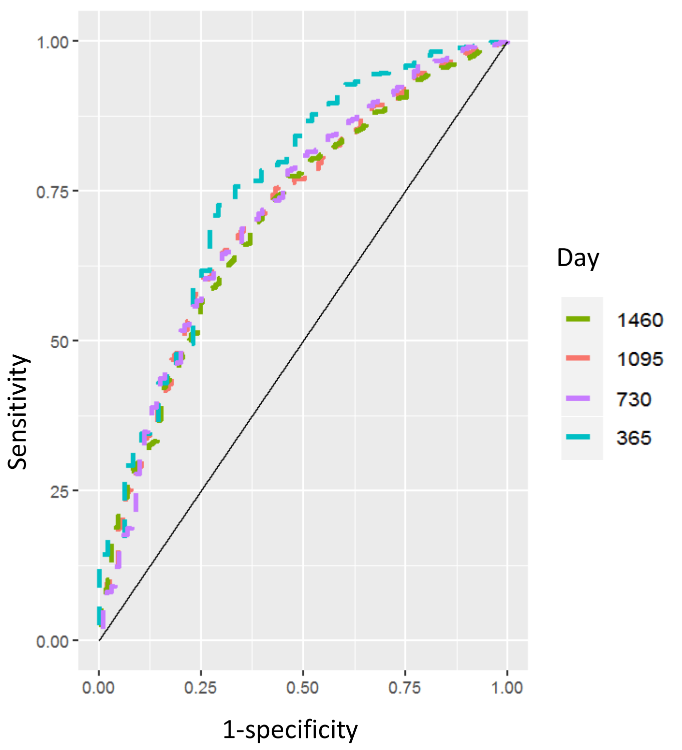

C. Dataset 3

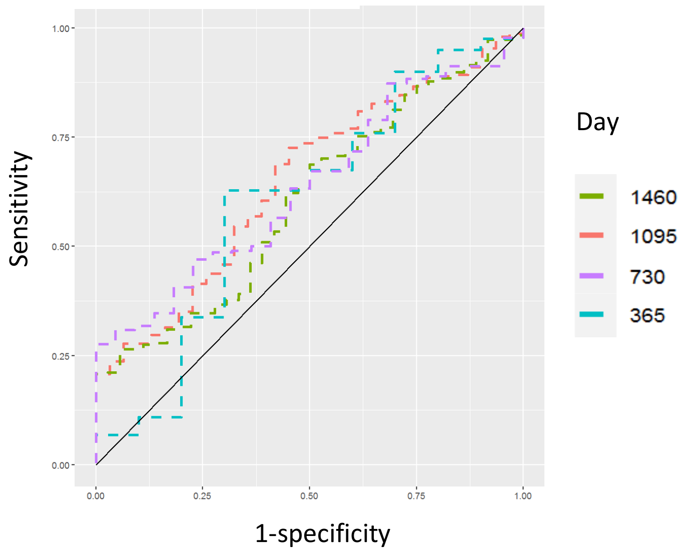

Supplementary Figure 5: ROC curve plotted for 365–1460 days after patient's first diagnosis date for dataset 1 (Figure 3A), dataset 2 (Figure 3B), and dataset 3 (Figure 3C). Sensitivity or true positive rate is shown on the *y* axis and 1 − specificity or false positive rate is shown on the *x* axis. The diagonal line shows prediction at chance.

Supplementary Figure 6: AUROC for SLaM data

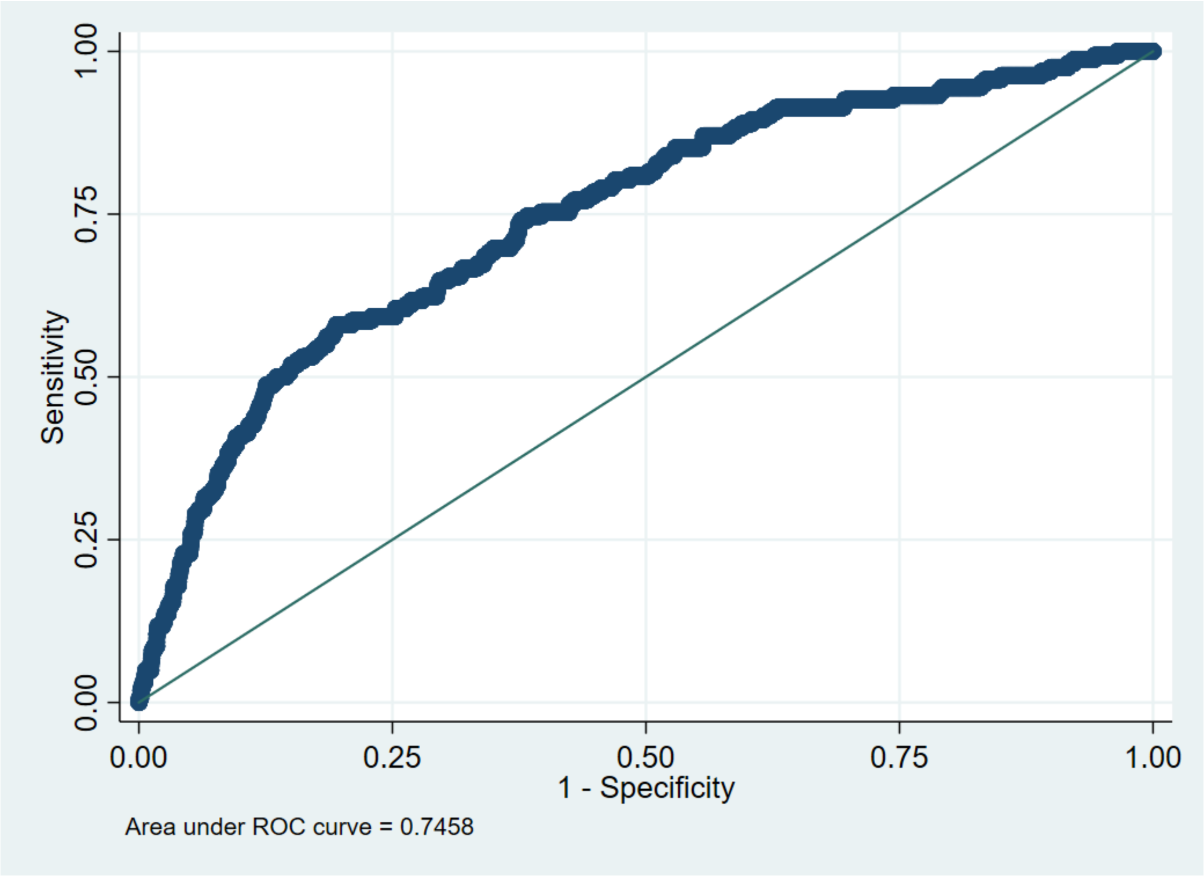

Supplementary Figure 6: AUROC for the SLaM dataset is 0.746. Sensitivity is shown on the *y* axis and 1 − specificity is shown on the *x* axis. The diagonal line shows prediction at chance.

Supplementary Figure 7: Dominance Analysis for CPFT Dataset (Dataset 1)

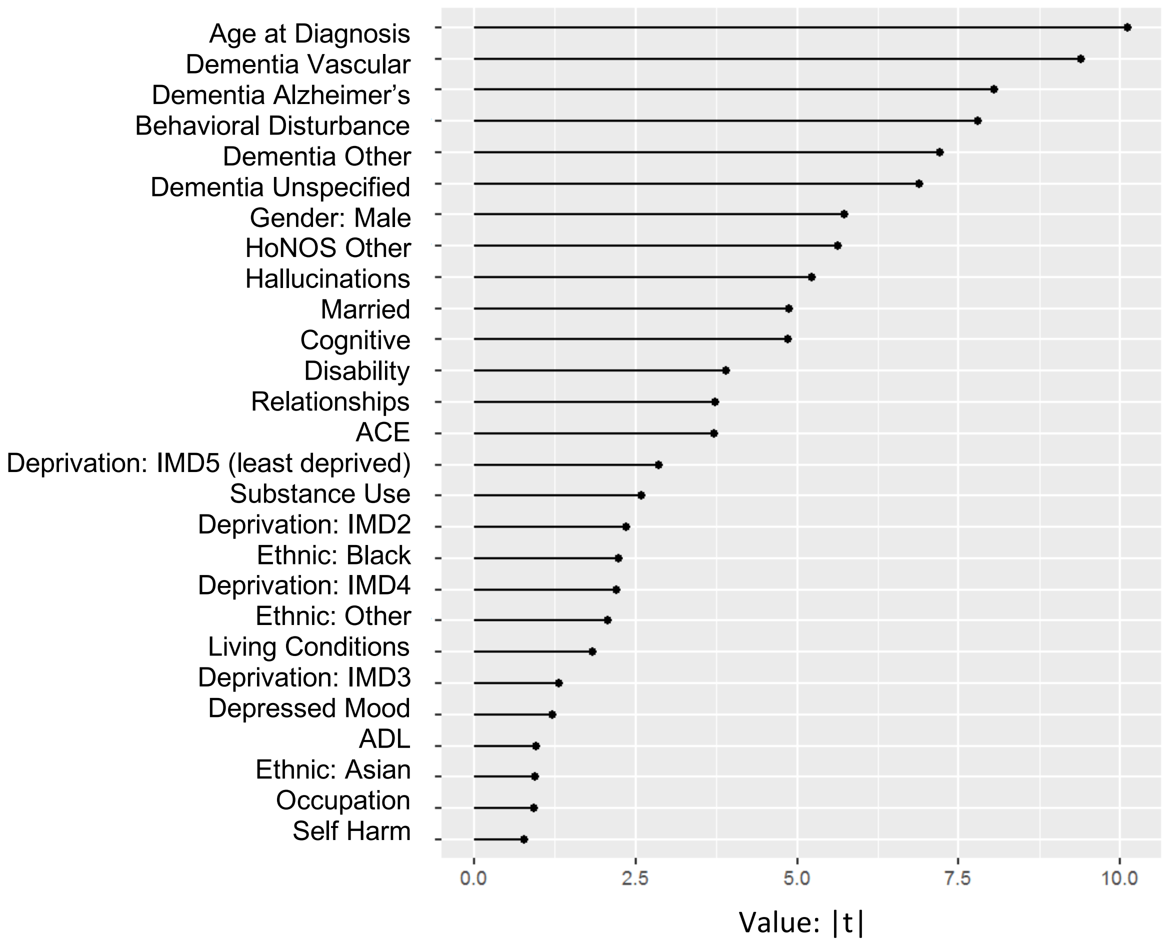

Supplementary Figure 7: Dominance analysis is shown above and lists the variables from most important to those that are least important in predicting patients who are admitted to the crisis or inpatient units. The most important variables were age, dementia subtype, and behavioural disturbance on the HoNOS. Variables which did not significantly predict outcome were ADL, ethnicity, occupation, and self harm. The value and ranking are based on the t-statistic.

Supplementary Table 4: Comparison of Patients Receiving and Not Receiving Enhanced Care in SLaM

Variable	Received crisis care (n=162)	No crisis care (n=6,567)	p-value[1]
Sociodemographic variables			
***Age at diagnosis (mean, SD)**	77.5 (8.2)	82.2 (7.7)	<1.00e-3
Female (%)	53.7%	61.0%	0.0620
Ethnicity			0.0580
White (%)	69.1%	70.0%	
Black (%)	26.5%	20.5%	
Asian (%)	3.10%	6.30%	
Other (%)	1.20%	3.20%	
****Married or cohabiting (%)**	43.8%	32.5%	2.00e-3
****Index of multiple deprivations (mean, SD)**	26.8 (10.1)	24.5 (10.1)	5.00e-3
Cognitive score / MMSE score (mean, SD)	17.9 (6.20)	17.4 (6.70)	0.377
Dementia subtype			0.115
Alzheimer's disease (F00)	78.4%	73.7%	
Vascular dementia (F01)	11.7%	15.2%	
Dementia in other diseases (F02)	5.60%	3.50%	
Unspecified dementia (F03)	4.30%	7.60%	
***Total HoNOS score[2]**	11.2 (5.20)	10.2 (5.30)	0.0120
Mental Health Problems according to HoNOS[2]			
*****Behaviour disturbance (%)**	28.4%	15.0%	<1.00e-3
Self Harm (%)	2.50%	1.12%	0.135
Substance use (%)	4.90%	3.10%	0.174
Cognitive problems (%)	86.4%	84.7%	0.550
****Hallucinations (%)**	19.1%	10.6%	1.00e-3
****Depressed mood (%)**	21.6%	13.5%	3.00e-3
*****Physical illness or disability according to HoNOS[2]**	40.1%	54.6%	<1.0e-3
Functional problems according to HoNOS[2]			
*****Relationships (%)**	24.7%	12.3%	<1.0e-3
ADL (%)	51.9%	58.1%	0.113
Living conditions (%)	12.4%	11.6%	0.776
Occupation/Activities (%)	30.3%	31.9%	0.658

Supplementary Table 4: Characteristics of those receiving vs not receiving crisis care including sociodemographic variables, dementia subtype, total HoNOS score, and HoNOS subscroes. Bold items are significant; *p <0.05, ** p <0.01, *** p <0.001. Three significant figures are used. [1]P values were calculated using a t-test or chi-square test. [2]The percent of patients with a Health of the Nation Outcome Scale (HoNOS) subscale score of >=2 is indicated in the table as this score was taken to indicate the presence of a problem.

Supplementary Table 5: Logistic Regression Model in SLaM (with odds ratios as output) Using HoNOS Subscales as Binary Variables (0-1: no problem; 2-4: problem present)

```
. logistic crisis_care Age_at_diagnosis Gender_code Marital_code i.Ethnicity_code IMD_score_2019 i.diag
> nosis_groups agait_prob selfinj_prob substanceuse_prob cognitive_prob depr_prob hallu_prob physical_p
> rob relat_prob ADL_prob livcon_prob occu_prob MMSE_Numerator

Logistic regression                             Number of obs     =      6,729
                                                LR chi2(22)       =     131.26
                                                Prob > chi2       =     0.0000
Log likelihood = -698.11087                     Pseudo R2         =     0.0859
```

crisis_care	Odds Ratio	Std. Err.	z	P>|z|	[95% Conf.	Interval]
Age_at_diagnosis	.9418686	.0093841	-6.01	0.000	.9236545	.9604419
Gender_code	.8841646	.1516425	-0.72	0.473	.6317483	1.237434
Marital_code	1.374466	.2372171	1.84	0.065	.9800005	1.92771
Ethnicity_code						
2	1.115676	.2142008	0.57	0.569	.7657976	1.625406
3	.3769693	.1755774	-2.09	0.036	.1513038	.9392085
4	.3859736	.2783383	-1.32	0.187	.0939135	1.586306
IMD_score_2019	1.020102	.0083449	2.43	0.015	1.003876	1.036589
diagnosis_groups						
2	.6336785	.1658491	-1.74	0.081	.3793925	1.058398
3	.8197287	.318656	-0.51	0.609	.3826305	1.756146
4	.4668423	.1882213	-1.89	0.059	.2118279	1.028862
agait_prob	1.844287	.4075592	2.77	0.006	1.195986	2.844009
selfinj_prob	1.436598	.7867347	0.66	0.508	.4911178	4.20228
substanceuse_prob	.9747891	.3787608	-0.07	0.948	.4551669	2.087616
cognitive_prob	1.29025	.3227351	1.02	0.308	.7902408	2.106629
depr_prob	1.574003	.330601	2.16	0.031	1.042847	2.375694
hallu_prob	1.7313	.3975374	2.39	0.017	1.103879	2.715335
physical_prob	.5439412	.1001892	-3.31	0.001	.3791125	.7804333
relat_prob	1.798767	.4032709	2.62	0.009	1.159155	2.791312
ADL_prob	.8363191	.1654947	-0.90	0.366	.5674562	1.23257
livcon_prob	.9711171	.257744	-0.11	0.912	.5772372	1.633762
occu_prob	.8339434	.1686274	-0.90	0.369	.5610732	1.23952
MMSE_Numerator	1.016667	.0132167	1.27	0.204	.9910904	1.042904
_cons	1.238759	1.135748	0.23	0.815	.205385	7.471455

Note: _cons estimates baseline odds.

Supplementary Table 5: Output of the logistic regression for SLaM data using HoNOS as binary variables. All the variables used in the model are shown above including odds ratio, 95% confidence interval, standard error, and p values. For ethnicity, white was used as the reference. Ethnicity code 1: white, ethnicity code 2: black, ethnicity code 3: Asian, ethnicity code 4: other. Diagnosis group 1: Alzheimer's, diagnosis group 2: Vascular dementia, diagnosis group 3: Dementia in other diseases, diagnosis group 4: unspecified dementia

Additional References

R Packages Used for Model Development

The following R packages were used: lubridate (1), mice (2), survival (3), boot (4), relaimpo (5), dominanceanalysis (6), caret (7) , pROC (8), doParallel (9), naivebayes (10), nnet (11), dplyr (12), magrittr (13), and tidyverse (14).

1. Grolemund G, Wickham H (2011). "Dates and Times Made Easy with lubridate." *Journal of Statistical Software*, **40**(3), 1–25. https://www.jstatsoft.org/v40/i03/.

2. van Buuren S, Groothuis-Oudshoorn K (2011). "mice: Multivariate Imputation by Chained Equations in R." *Journal of Statistical Software*, **45**(3), 1-67. doi:10.18637/jss.v045.i03.

3. Therneau T (2024). *A Package for Survival Analysis in R*. R package version 3.5-8, https://CRAN.R-project.org/package=survival.

4. Angelo Canty, B. D. Ripley (2024). *boot: Bootstrap R (S-Plus) Functions*. R package version 1.3-30, https://cran.r-project.org/web/packages/boot/citation.html.

5. Groemping U (2006). "Relative Importance for Linear Regression in R: The Package relaimpo." *Journal of Statistical Software*, **17**(1), 1–27.

6. Azen, R., & Budescu, D. V. (2003). The dominance analysis approach for comparing predictors in multiple regression. *Psychological Methods, 8*(2), 129–148. https://doi.org/10.1037/1082-989X.8.2.129

7. Kuhn, Max (2008). "Building Predictive Models in R Using the caret Package." *Journal of Statistical Software*, **28**(5), 1–26. doi:10.18637/jss.v028.i05, https://www.jstatsoft.org/index.php/jss/article/view/v028i05.

8. Robin X, Turck N, Hainard A, Tiberti N, Lisacek F, Sanchez J, Müller M (2011). "pROC: an open-source package for R and S+ to analyze and compare ROC curves." *BMC Bioinformatics*, **12**, 77.

9. Weston, Steve, and Rich Calaway. *Getting Started with doParallel and Foreach*, Jan. 2022.

10. Majka M (2019). *naivebayes: High Performance Implementation of the Naive Bayes Algorithm in R*. R package version 0.9.7, https://CRAN.R-project.org/package=naivebayes

11. Venables WN, Ripley BD (2002). *Modern Applied Statistics with S*, Fourth edition. Springer, New York. ISBN 0-387-95457-0, https://www.stats.ox.ac.uk/pub/MASS4/

12. Wickham H, François R, Henry L, Müller K, Vaughan D (2023). *dplyr: A Grammar of Data Manipulation*. R package version 1.1.4, https://github.com/tidyverse/dplyr, https://dplyr.tidyverse.org.

13. Bache S, Wickham H (2022). *magrittr: A Forward-Pipe Operator for R*. https://magrittr.tidyverse.org, https://github.com/tidyverse/magrittr.

14. Wickham H, Averick M, Bryan J, Chang W, McGowan LD, François R, Grolemund G, Hayes A, Henry L, Hester J, Kuhn M, Pedersen TL, Miller E, Bache SM, Müller K, Ooms J, Robinson D, Seidel DP, Spinu V, Takahashi K, Vaughan D, Wilke C, Woo K, Yutani H (2019). "Welcome to the tidyverse." *Journal of Open Source Software*, **4**(43), 1686. doi:10.21105/joss.01686.
